# The Prevalence of HIV by Ethnic Group Is Correlated with HSV-2 and Syphilis Prevalence in Kenya, South Africa, the United Kingdom, and the United States

**DOI:** 10.1155/2014/284317

**Published:** 2014-09-24

**Authors:** Chris Richard Kenyon, Kara Osbak, Jozefien Buyze

**Affiliations:** ^1^Sexually Transmitted Infections, HIV/STI Unit, Institute of Tropical Medicine, Antwerp, Belgium; ^2^Division of Infectious Diseases and HIV Medicine, University of Cape Town, Anzio Road, Observatory 7700, South Africa; ^3^HIV/STI Unit, Institute of Tropical Medicine, Antwerp, Belgium

## Abstract

*Background*. This paper investigates two issues: do ethnic/racial groups with high HIV prevalences also have higher prevalences of other STIs? and is HIV prevalence by ethnic group correlated with the prevalence of circumcision, concurrency, or having more than one partner in the preceding year? *Methods*. We used Spearman's correlation to estimate the association between the prevalence of HIV per ethnic/racial group and HSV-2, syphilis, symptoms of an STI, having more than one partner in the past year, concurrency, and circumcision in Kenya, South Africa, the United Kingdom, and the United States. *Results*. We found that in each country HSV-2, syphilis, and symptomatic STIs were positively correlated with HIV prevalence (HSV-2: Kenya rho = 0.50, *P* = 0.207; South Africa rho-1, *P* = 0.000; USA rho-1, *P* = 0.000, Syphilis: Kenya rho = 0.33, *P* = 0.420; South Africa rho-1, *P* = 0.000; USA rho-1, *P* = 0.000, and STI symptoms: Kenya rho = 0.92, *P* = 0.001; South Africa rho-1, *P* = 0.000; UK rho = 0.87, *P* = 0.058; USA rho-1, *P* = 0.000). The prevalence of circumcision was only negatively associated with HIV prevalence in Kenya. Both having more than one partner in the previous year and concurrency were positively associated with HIV prevalence in all countries (concurrency: Kenya rho = 0.79, *P* = 0.036; South Africa rho-1, *P* = 0.000; UK 0.87, *P* = 0.058; USA rho-1, *P* = 0.000 and multiple partners: Kenya rho = 0.82, *P* = 0.023; South Africa rho-1, *P* = 0.000; UK rho = 0.87, *P* = 0.058; USA rho-1, *P* = 0.000). Not all associations were statistically significant. *Conclusion*. Further attention needs to be directed to what determines higher rates of partner change and concurrency in communities with high STI prevalence.

## 1. Introduction

The spread of HIV through populations around the world was remarkably uneven. Of the 149 countries for which the WHO provides data, 98 had a peak HIV prevalence that never went above 1% and only in 20 countries peak HIV prevalence exceeded 5% [1]. So too was the spread of HIV within countries uneven. In Kenya, South Africa (SA), and the United States of America (USA), for example, HIV prevalence varied by a factor of 20, 40, and 7.8, respectively, between the racial/ethnic groups within these countries [2–4].

There is still considerable debate about what determines these differences. A number of studies investigating this issue have utilized individual level risk factor study designs [5, 6]. Since HIV is spread over networks of sexual relationships, individual level studies miss the contribution played by network level determinants [7]. Ecological level studies are a valid way to assess network level factors [8]. The ecological level studies that have been performed have however generally been limited to one or two countries [2, 3, 9]. The weakness of this study design is that any association found may represent confounding. In this paper we have two aims. Firstly, we aim to assess in four countries (Kenya, SA, United Kingdom (UK), and USA) if any of the three risk factors (the prevalence of concurrency, having more than one sexual partner per year, and circumcision) is associated with HIV prevalence by ethnic group in each of these countries.

Secondly, we assess if ethnic groups more heavily affected by HIV have higher rates of other STIs preceding the HIV epidemic. The answer to this question has significant consequences. If there was no correlation between HIV and STI prevalence then the affected populations likely suffer from some factor that makes them specifically more vulnerable to HIV. This would give more credence to HIV prevention strategies that focused on HIV specific responses such as antiretroviral treatment as prevention [10]. If however there was a correlation this would suggest that local sexual networks were more generally conducive to the spread of STIs (including HIV) in populations with high HIV prevalences. If this was true, then it would make more sense for STI prevention to focus on dealing with this general STI enhancing transmission factor(s). The four countries were selected on the basis of their constitutive ethnic groups having wide variations in HIV prevalence and the availability of nationally representative data for sexual behavior and STI prevalence.

## 2. Methods

All the data used were taken from published studies. The individual studies providing this data are described in Table 1. The studies were selected as follows. Wherever possible surveys that were designed to provide samples that were representative for the country and individual ethnic groups were used. Where this was not possible we used large samples such as antenatal surveys that would provide a close approximation to representative samples. If a number of studies fulfilled our criteria then we chose the earliest sample for the studies reporting the prevalence of sexual behaviours, circumcision, and STIs and the most recent estimates for HIV prevalence. The reasons for this choice of timing were that there is evidence that sexual behaviours have changed in response to the HIV epidemic and the magnitude of this change may be correlated with how the population was affected by HIV [2, 11, 12]. Since we wanted to assess if sexual risk factors early on or before the HIV epidemic were correlated with HIV prevalence we chose the earliest measures we could find. In the case of syphilis the earliest high quality prevalence data we could find was from 1976 to 1980 for the USA and 1991 for South Africa [13, 14]. For HSV-2, we could only find early prevalence data from one country—the USA in 1976−1980 and 1988–1994 [15]. There is evidence that HIV may increase the transmission of certain STIs such as HSV-2 [16] and, via a number of distinct mechanisms, decrease the transmission of others such as syphilis [17, 18]. To avoid confounding by HIV prevalence, we used the earliest available representative STI prevalence data. Since the spread of HIV may take off at different time points, it makes most sense to use peak HIV prevalence to compare the HIV epidemics in different ethnic groups [19]. This correlates most closely with the more recent estimates of HIV prevalence [1]. HSV-2 and syphilis were chosen as they are amenable to serological assessment in large population-based surveys.

*Kenya*. The only nationally representative surveys which have measured HSV-2 and syphilis seroprevalence did not collect data on the ethnicity of the respondents. Ethnic group membership in Kenya is however highly correlated with region of residence [20]. For example, 75.1% of the Luo and 76.3% of the Kisii live in Nyanza Province where they make up 60.1 and 32.1% of the population, respectively (authors calculations of Kenya Demographic and Health Survey 2008 data [4]). As a result a number of epidemiology studies have used regions as proxies for ethnicity [6, 21]. We follow this approach and use the Kenya 2007 AIDS Indicator Survey to provide prevalence estimates for HSV-2 and syphilis by region [22].

### 2.1. Variables

The prevalence of* concurrency* was defined as the proportion of men (15–49 years old) who reported having two or more sex partners at the time of the survey, except in the UK where it refers to the cumulative prevalence of calculated concurrency over the prior year. In the UK, partnerships were classified as concurrent if the month and year of the first sex with the more recent partner were before the month and year of the last sex with the former partner.

The prevalence of* multiple partners* was defined as the percentage of men (15–49 years old) who reported having 2 or more sex partners in the last 12 months. This variable was not reported by ethnic/racial group in the UK surveys and thus here we used the percent of men who reported one or more new heterosexual relationship in the prior year.

The prevalence of* circumcision* refers to the percent of men who reported themselves having undergone circumcision.

*HIV* prevalence was defined as the percent of men and women (15–49 years old) who tested positive for HIV.

The prevalence of* syphilis* is defined as the percentage of men and women (15–49 years old) who tested positive for syphilis. This was assessed using different methodologies, as described in Table 1.

All* HSV-2* prevalence estimates were assessed using HSV-2 specific antibody tests and refer to the combined male and female prevalence.

*Males Reporting STIs. Male urethral discharge syndrome (MUDS)* was defined as the percent of all men, 15–49 years old, who reported having experienced symptoms of urethral discharge in the previous 3 months (SA) or 12 months (Kenya).

In the case of the UK and USA the proportion reporting MUDS was not available. For the UK and USA we used the proportion of men who reported having ever before been diagnosed with an STI (UK) or gonorrhoea (USA).

All the variables were limited to the 15–49-year-old age group except

1. the circumcision and sexual behavioural data from the USA which was reported in 18–59-year olds;
2. the HSV-2 data from the USA in 1976–1980 and 1988–1994 which included all those who aged 12 years or older;
3. the sexual behavior data from the UK referring to 16–44-year olds;
4. HSV-2 and syphilis prevalence in Kenya referring to 15–64-year olds.

### 2.2. Statistical Analysis

The small number of ethnic/racial groups per country makes the use of statistical tests of correlation of questionable merit. Nonetheless we investigated the statistical significance of the correlations between HIV and the other STIs and risk factors using Spearman's correlation coefficient. We chose Spearman's correlation coefficient for these analyses as the sample sizes were small and in a number of cases the relationship between the variables was nonlinear. The analysis was performed using STATA 12 software (Stata, East College Station, TX, USA). Where appropriate, the SVY function was used to adjust for complex survey designs and differential nonresponse rates. Because of the small number of ethnic/racial groups per country, a *P* value below 0.1 was considered statistically significant.

## 3. Results

### 3.1. Variations in STI Prevalence

**Figure 1:**
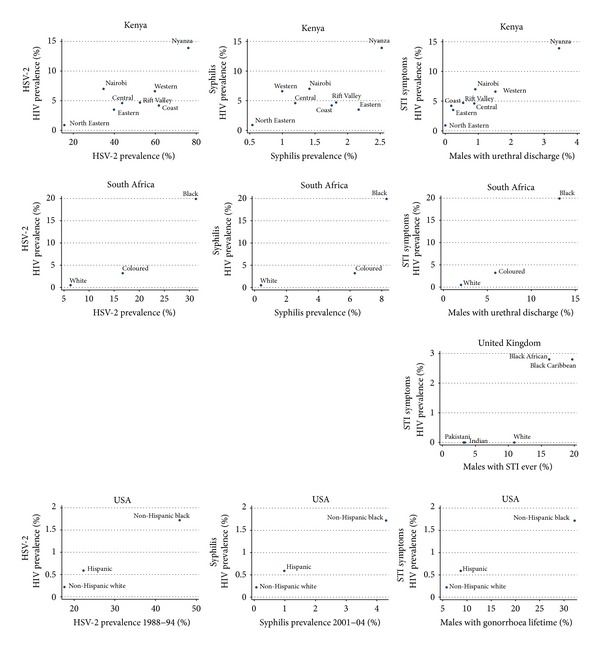
The prevalence of HSV-2, syphilis, and symptomatic STI versus HIV prevalence by ethnic group in Kenya, South Africa, the United Kingdom, and the United States of America.

**Figure 2:**
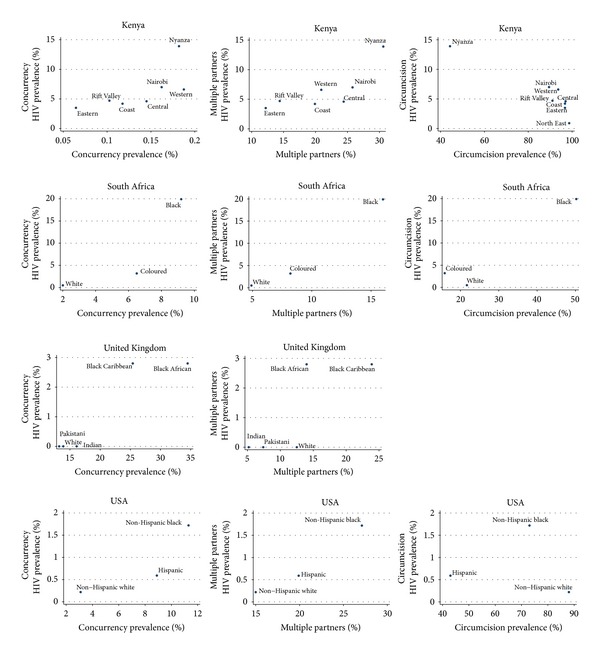
The prevalence of concurrency, multiple partnering, and circumcision versus HIV prevalence by ethnic group in Kenya, South Africa, the United Kingdom, and the United States of America.

The relative risk of HIV by ethnic/racial group varied between 7.8 and 39.8 within each country (for prevalences of each STI see Table 2 and Figures 1 and 2). The corresponding figures varied from 5.0- to 61.4-fold for syphilis, 2.6- to 4.9-fold for HSV-2, and 5.6- to 19.2-fold for males reporting STIs.

### 3.2. Correlation between STIs and HIV

All three STI categories were positively associated with HIV prevalence in all countries (see Table 3 and Figure 1). Using a significance level of *P* < 0.1, this relationship was only statistically significant in South African and USA data from 1988 to 1994. The strength of Spearman's correlation coefficient varied from 0.33 to 1. The data from the USA covered the longest period (32 years) and showed little change in the magnitude of the relative or absolute difference in HSV-2 prevalence between the non-Hispanic blacks and whites. Differences in the way Hispanics were defined between surveys make longitudinal prevalence estimates of this group less comparable [15, 23]. HSV-2 prevalence estimates from the first survey (1976) were limited to the non-Hispanic whites (12.7%) and the non-Hispanic blacks (43.6%) [15]. In the 1988–1994 and 2005–2008 surveys, except for the Hispanics, there was little relative change in HSV-2 prevalence of non-Hispanic whites, Hispanics, and non-Hispanic blacks (17.6%, 22.3%, and 45.9%, respectively, in 1988–1994 and 12.3%, 10.1%, and 39.2% in 2005–2008).

Although the relationship between HIV and syphilis rates was positive in the three countries assessed, this relationship was only statistically significant in South Africa and the USA (Spearman's correlation coefficient = 1.0; *P* = 0.000 for both countries). The relationship between symptomatic STI and HIV prevalence was positive and statistically significant in all four countries (Spearman's correlation coefficient ranging from 0.87 to 1.0).

### 3.3. Variations in the Prevalence of Risk Factors

There were large variations in the prevalence of circumcision, multiple partnering, and concurrency by ethnic group. The prevalence of circumcision by ethnic group in USA, SA, and Kenya varied by a factor of up to 1.2, 3.2, and 2.2, respectively. The prevalence of multiple partnering in the USA, SA, Kenya, and the UK varied by a factor of up to 1.8, 3.3, 2.5, and 1.9, respectively. Concurrency prevalence in the same countries varied by a factor of 3.6, 4.6, 2.9, and 2.6, respectively.

### 3.4. Correlation between Sexual Behaviors and HIV

There was no statistically significant correlation between circumcision and HIV prevalence in any of Kenya, SA, or USA. The prevalence of circumcision by race in the NATSAL surveys from the UK was not reported but it was stated in the report that, “with the exception of black Caribbeans, men from all ethnic minority backgrounds were significantly more likely to report being circumcised compared to men who described their ethnicity as white (adjusting for various demographic variables) (adjusted odds ratio (OR) 3.02, 95% CI 2.39 to 3.81)” [24].

The prevalence of multiple partnering and concurrency were positively associated with HIV prevalence in all countries. This association was statistically significant in all cases (Spearman's correlation coefficient ranging from 0.79 and 1.0).

## 4. Discussion

In all four countries HIV prevalence was positively correlated with the prevalence of HSV-2, syphilis, and males reporting an STI. This positive association applied to the pre-HIV and HIV periods. In the case of HSV-2 in the USA, there was little change in the relative differences in HSV-2 prevalence between the various racial/ethnic groups over a 32-year period. Thus HSV-2 prevalence in non-Hispanic black women was as high in 1976 (51%) as in 2008 (48%). Studies that control differences such as age structures between the different surveys have found that there has been a small increase in the non-Hispanic black to white ratio of syphilis over this time period [25]. Likewise, in one of the few other locales to have representative HSV-2 prevalence data from an extended period, a study from Northern Malawi found that HSV-2 prevalence did not increase from 1988 to 2005, a time when adult HIV prevalence increased from 4% to 12% [26].

In SA the earliest comparison of HSV-2 prevalence by racial group was a survey of blood bank donors in 2005 [27]. In this survey, the HSV-2 prevalences were lower than those taken from the 2012 antenatal survey, but the HSV-2 prevalence was five times higher in the blacks than the whites. This was slightly higher than the three-fold higher rate seen in the 2012 antenatal survey.

A strong association between HIV and HSV-2 has also been found at the level of countries and world regions. A study of 52 countries found a strong correlation between peak HIV and HSV-2 prevalence in 40–44-year-old women (Spearman's correlation coefficient = 0.720; *P* = 0.0001) [28]. So too, at the level of world regions it was found that there was a close association between HIV and HSV-2 prevalence [29].

We also found associations between HIV and syphilis and HIV and males reporting STIs. Similar associations have been found at the country and world region levels between the prevalence of syphilis before the HIV epidemic and peak HIV prevalence [18, 29].

Four factors suggest that the association between HIV and other STIs is due to factors which act as general STI transmission enhancing factors. Firstly, the association between HIV and other STIs has been found at different levels of aggregation (by ethnic group within countries, cross country, and between world regions) [2, 30–32]. Secondly, STI prevalence from before the advent of HIV was correlated with subsequent HIV prevalence suggesting that some factor predisposed to the spread of HIV and the other STIs. Thirdly, since this association between STIs and peak HIV prevalence applies to a range of viral [28, 29], bacterial [3, 18, 29, 33], eukaryotic [34], and polymicrobial infections [35], it is less likely that it is due to biological increased susceptibility to each of these very different categories of microbes. Fourthly, the way that these large differences in STI prevalence are found within Kenya's ethnic groups—all of which are black African—suggests that racial differences in susceptibility do not play a major role.

We examined three possible risk factors. Various pieces of evidence, including those presented here, suggest that differences in circumcision rates play a role in determining the more extensive HIV epidemic in the Luo in Nyanza Province in Kenya, than other groups [36]. There was however no evidence in our study that circumcision was playing a large role in influencing STI rates elsewhere. There was no significant association between HIV and circumcision prevalence in SA and the USA and a positive association in the UK.

Although this study only includes four countries both multiple partnering and concurrency were positively associated with HIV prevalence in all countries. Little more can be concluded from this other than this finding is congruent with findings elsewhere that populations with higher rates of partner change and concurrency have a higher prevalence of HIV and other STIs [2, 3, 8].

There are however a number of serious limitations to this study. Only three risk factors are investigated which means that our results could represent confounding. A variety of socioeconomic factors have been shown to be important in the genesis of differential STI rates by race/ethnic group in the USA [37, 38]. Although we did not control any socioeconomic differences in our study other studies from Sub-Saharan Africa have found a different relationship between HIV and factors such as income in this region as compared to those found in the USA [39]. Thus in a number of longitudinal and cross-sectional studies from a range of countries, HIV incidence and prevalence were found to be higher in higher socioeconomic strata [40, 41]. There was no significant association between the prevalence of poverty and HIV by ethnic group in Kenya [36]. The earliest variables we could find for some of the countries (such as HSV-2, syphilis, and male reported STIs from Kenya) were derived from surveys quite late in the HIV epidemic. As such they could have been affected by the HIV epidemic. In addition we used a *P* value threshold of 0.1 to assess the statistical significance. Using a lower threshold of 0.05 would have resulted in three associations (all from the UK) no longer being classified as being significant—STI symptoms versus HIV, concurrency versus HIV, and multiple partners versus HIV.

The prevalence of an STI in a particular community represents the composite outcome of the interaction of a number of different risk factors [42]. It is likely that the particular configuration of these risk factors will vary in different communities. Nonetheless if one finds certain factors to be associated with the prevalence of a range of STIs, in a range of different countries and at different levels of aggregation, then this provides suggestive evidence that they play a role in STI spread. If so then this provides information that can be fed back to affected communities to motivate behavior change. More work is, however, required to extend this type of analysis to include more risk factors, other STIs, more local and global levels of aggregation, and over longer time periods.

## Figures and Tables

**Table 1:** Description of sources of data for prevalence of STIs, multiple partners, concurrency, and circumcision.

	Year in which data were collected, reference	Study type, selection procedure, sample size, response rate, and testing protocol for syphilis
*South Africa *		
HIV	2005 [12]	A two-stage, nationally representative sample of 23,275 persons 2 years old or older. We limited our analysis to the 13,884 individuals aged 15 to 49 years old. The survey had an overall response rate of 80.7%
HSV-2	2012 [43]	A sample of 18,732, 15–49-year-old women attending antenatal clinics for the first time in four provinces (Western Cape, Northern Cape, Gauteng, and Kwazulu Natal) were tested for HSV-2. Response rate not reported
Syphilis	1991 [14]	A sample of 17,318, 15–49-year-old women attending antenatal clinics for their first visit were tested for syphilis via the Rapid Plasma Reagin or the Venereal Diseases Research Laboratory test. Response rate not reported. The unadjusted prevalence estimates were used
Urethral discharge	1998 [44]	South Africa's first Demographic and Health Survey employed a 2-stage sampling strategy in South Africa's nine provinces and stratified results into urban and nonurban groups. It was designed to be representative of all provinces and the four major racial groups. 6,578 men were asked if they had experienced symptoms of a urethral discharge in the last 3 months. The overall response rate for men was 89.7%. Men were not asked questions about their sexual behavior in this survey
Concurrency, multiple partners, and circumcision	2003 [45]	The 2003 Demographic Health Survey (DHS) used a similar study design to the 1998 DHS. The survey sampled 7,966 women and 3,930 men. All were 15–49 years old [45]. The overall response rate for men was 67.8%

*USA *		
HIV	2006 [46]	HIV prevalence at the end of 2006 was estimated using information from the national HIV/AIDS reporting system
HSV-2	1988–94 [15]	In NHANES III, a national stratified probability sample of 13,094 individuals over the age of 12 were tested for HSV-2. These persons comprised 60.5% of all respondents in NHANES III
	2005–09 [23]	During NHANES 2005–2008, a total of 8,283 persons aged 14–49 years were interviewed. Of these, 7,293 participants (88% of those interviewed) were tested for HSV-2 antibodies
Syphilis	1976–80 [13]	In NHANES II a national stratified probability sample of 12,989 individuals over the age of 12 were tested for syphilis. 92% of those who were examined provided blood for syphilis testing. The initial test was with an RPR and confirmation with a microhemagglutination assay for *Treponema pallidum* (MHA-TP) or the fluorescent treponemal antibody absorption test (FTA-ABS)
	2001–2004 [47]	Sera from 5,767, 18- to 49-year-old participants in the NHANES 2001–2004, were tested for syphilis IgG antibody using an enzyme immunoassay (EIA). Specimens with positive or indeterminate EIAs underwent rapid plasma reagin (RPR) testing
Concurrency and multiple partners	1992 [48]	The USA **sexual behaviour data** and **gonorrhoea incidence** data were taken from the 1992 National Health and Social Life Survey (NHSLS). This was a cross-sectional study that used a nationally representative stratified random sample of 3,432 women and men between the ages of 18 and 59. The overall response rate was 78.6%
Circumcision	1999–2004 [49]	As part of National Health and Nutrition Examination Surveys from 1999 to 2004, 6,174 men were interviewed about circumcision status. The response rate was 86%

*Kenya *		
HIV, MUDS, and circumcision	2008 [20]	The 2008 Kenya Demographic and Health Survey used a household-based, two-stage stratified sampling approach to recruit 12,677 participants. The overall household response rate was 97.7%
HSV-2 and syphilis	2007 [22]	The 2007 Kenya AIDS Indicator Survey used a stratified two-stage sampling strategy to test a nationally and provincially representative sample of 15,853, 15–64-year olds. Syphilis was screened for via a *Treponema pallidum *particle agglutination assay (TPPA) (Serodia-TPPA, Fujirebio Diagnostics Inc.). All TPPA positive specimens were tested using the rapid plasma regain (RPR) (Macro-Vue RPR Card Test, BD, USA) on undiluted serum. Only those positive on both TPPA and RPR were classified as syphilis
Concurrency and multiple partners	2011 [50]	The Population Services International (PSI) Survey/Kenya 6th HIV Survey conducted in 2011 used a two-stage cluster sampling to obtain a provincially representative sample of households from seven of Kenya's eight provinces (the North East was excluded). A total of 3,051 men and women, 15–49 years old, were included. Response rate not reported

*United Kingdom *		
HIV and MUDS	2010–2012 [51]	National Surveys of Sexual Attitudes and Lifestyles 3 recruited a probability sample of 15,162 women and men aged 16–74 years in Britain. Participants were interviewed with computer-assisted face-to-face and self-completion questionnaires. Urine from a sample of participants aged 16–44 years who reported at least one sexual partner over the lifetime was tested for HIV antibodies
Concurrency, multiple partners, STI, and circumcision	2000 [24, 52]	The second British National Survey of Sexual Attitudes and Lifestyles (NATSAL 2) was a nationally representative sample of 11,161 men and women aged 16–44 years [52]. We extracted the relevant variables from a study which broke down the various sexual behavior variables by ethnic group and sex [52]. Men were asked if they had ever been diagnosed with an STI

DHS: Demographic and Health Survey, NATSAL: National Survey of Sexual Attitudes and Lifestyles, NHANES: National Health and Nutrition Examination Surveys.

**Table 2:** The prevalence of HIV, syphilis, HSV-2, STI symptoms, concurrency, multiple partners, and circumcision by ethnic/racial group.

Country	Ethnic/racial group	HIV	Syphilis (early)^a^	Syphilis (late)^b^	HSV-2 (early)^c^	HSV-2 (late)^d^	STI symptoms	Concurrency	Multiple partners	Circumcision
USA	Non-Hispanic white	0.22 (0.21–0.24)	0.53 (0.39–0.67)	0.07 (0.01–0.28)	17.6 (15.7–19.8)	12.3 (10.7–14.2)	5.8 (4.6–7.3)	3.1 (2.2–4.3)	15.0 (14.0–16.5)	88^e^
	Hispanic	0.59 (0.53–0.64)		0.98 (0.49–1.74)	22.3 (21.2–23.5)	10.1 (8.3–12.)	8.7 (2.7–17.8)	8.9 (3.6–19.9)	19.9 (15.7–24.7)	
	Non-Hispanic black	1.72 (1.61–1.82)	3.05 (2.18–3.92)	4.3 (3.23–5.53)	45.9 (43.9–47.9)	39.2 (36.7–41.7)	32.2 (25.9–39.0)	11.3 (7.5–16.7)	27.1 (23.8–31.4)	73^e^

SA	White	0.5 (0.3–1.1)	0.4 (0.1–0.9)			19.5 (12.7–28.2)	2.0 (0.7–3.2)	2.0 (0.3–12.5)	4.9 (2.1–11.2)	21.7 (13.8–32.5)
	Coloured	3.2 (2.3–4.6)	6.3 (5.4–7.3)			30.3 (28.1–32.5)	5.9 (4.2–7.5)	6.5 (2.7–14.8)	8.2 (4.9–13.6)	15.9 (10.6–23.0)
	Black	19.9 (18.3–21.7)	8.3 (7.8–8.8)			60.8 (60.1–61.5)	13.2 (12.2–14.2)	9.2 (7.1–12.0)	16 (13.9–18.3)	50.2 (47.3–53.7)

Kenya	Nairobi	7.2 (4.2–12.2)		1.4 (0.8–2.1)		34.7 (32.3–37.3)	0.91 (0.23–3.5)	16.0 (11.7–20.8)	25.8 (19.8–32.9)	89.5 (81.1–94.4)
	Central	4.6 (3.2–6.8)		1.2 (0.7–1.8)		43.8 (41.1–46.5)	0.88 (0.22–3.3)	14.5 (11.1–18.4)	24.4 (17.3–33.3)	97.0 (94.1–98.5)
	Coast	4.2 (2.6–7.0)		1.8 (1.1–2.7)		61.8 (58.9–64.6)	0.18 (0.04–0.77)	11.7 (8.5–15.6)	19.9 (14.2–27.3)	96.7 (92.7–98.6)
	Eastern	3.5 (2.1–5.5)		2.2 (1.5–3.1)		39.9 (37.3–42.5)	0.24 (0.05–1.0)	6.4 (4.2–9.2)	12.2 (7.4–19.5)	96.8 (93.6–98.4)
	Nyanza	13.9 (11.0–17.9)		2.5 (1.8–3.4)		76.2 (73.9–78.3)	3.45 (2.0–5.8)	18.2 (14.9–21–9)	30.6 (23.8–38.3)	44.3 (32.6–56.8)
	Rift Valley	4.7 (3.1–7.3)		1.8 (1.2–2.6)		52.6 (50.1–55.1)	0.54 (0.2–1.6)	10.3 (7.6–13.5)	14.4 (10.5–19.4)	91.1 (86.3–94.3)
	Western	6.6 (4.9–9.2)		1.0 (0.5–1.7)		59.9 (57.0–62.6)	1.52 (0.7–3.2)	18.8 (14.0–24.3)	20.9 (18.4–23.7)	93.7 (88.5–96.6)
	North Eastern	0.9 (0.3–4.0)		0.5 (0.2–1.4)		15.6 (12.9–18.5)	0 (0-0)	—	—	98.7 (96.1–99.6)

UK	White	0^e^					10.9 (9.9–12.0)	13.9 (12.7–15.3)	29.6 (28.1–31.1)	
	Black Caribbean	2.8					19.7 (13.6–27.9)	25.4 (17.6–35.1)	41.6 (31.3–52.7)	
	Black African	2.8					16.2 (10.8–23.4)	34.5 (22.8–48.3)	43.8 (34.1–54.1)	
	Indian	0					3.4 (1.0–11.9)	16.1 (8.0–29.8)	23.2 (16.5–31.5)	
	Pakistani	0					3.2 (1.3–7.5)	13.2 (6.9–23.7)	22.7 (14.8–33.2)	

^a^USA data from 1976 to 1980 [13]; SA data from 1991 [14].

^b^USA data from 2001–2004 [47]; Kenyan data from 2007 [22].

^c^USA data from 1988–1994 [15].

^d^USA data from 2005 to 2009 [23]; SA data from 2012 [43]; Kenyan data from 2007 [22].

^e^Confidence intervals were not specified.

**Table 3:** Spearman's correlation coefficient (rho) for the relationship between HIV prevalence and various risk factors and other STIs by racial/ethnic group in Kenya, South Africa, the United Kingdom (UK), and the United States of America (USA).

	Kenya		South Africa		UK		USA	
	Spearman's coefficient	*P*	Spearman's coefficient	*P*	Spearman's coefficient	*P*	Spearman's coefficient	*P*
HSV-2 (early)							1.0	0.000
HSV-2 (late)	0.50	0.207	1.0	0.000			0.50	0.667
Syphilis	0.33	0.420	1.0	0.000			1.0	0.000
STI symptoms	0.92	0.001	1.0	0.000	0.87	0.058	1.0	0.000
Concurrency	0.786	0.036	1.0	0.000	0.87	0.058	1.0	0.000
Multiple partners	0.821	0.023	1.0	0.000	0.87	0.058	1.0	0.000
Circumcision	−0.05	0.215	0.5	0.667			−0.50	0.667
